# Separate episodes of capillary leak syndrome and pulmonary hypertension after adjuvant gemcitabine and three years later after nab-paclitaxel for metastatic disease

**DOI:** 10.1186/1471-2407-13-542

**Published:** 2013-11-12

**Authors:** Andrea Casadei Gardini, Michele Aquilina, Devil Oboldi, Alessandro Lucchesi, Silvia Carloni, Elena Tenti, Marco Angelo Burgio, Dino Amadori, Giovanni Luca Frassineti

**Affiliations:** 1 Istituto Scientifico Romagnolo per lo Studio e la Cura dei Tumori (IRST) IRCCS, Via Piero Maroncelli 40, 47014 Meldola, FC, Italy

**Keywords:** Capillary leak syndrome, Chemotherapy, Gemcitabine, Nab-paclitaxel, Abraxane, Pulmonary hypertension

## Abstract

**Background:**

Systemic capillary leak syndrome is a rare disease with a high mortality rate. This syndrome is characterised by generalised edema, hypotension, hemoconcentration, and hypoproteinemia. The cause is the sudden onset of capillary hyperpermeability with extravasations of plasma from the intravascular to the extravascular compartment. We present the case of a patient who experienced two episodes of systemic capillary leak syndrome and pulmonary hypertension; the first after gemcitabine in an adjuvant setting and the second three years later after treatment with nab-paclitaxel for metastatic disease.

**Case presentation:**

A 65-year-old patient underwent a pancreatectomy in January 2010 for ductal carcinoma (pT3 N0 M0, stage IIa), followed by adjuvant chemotherapy. Seven days after the last cycle, she developed dyspnea associated with orthopnea and cough. A transthoracic cardiac ecocolordoppler was performed, with evidence of pulmonary hypertension (58 mmHg). Blood tests showed an increase in creatinine, pro-BNP and D-Dimer. She began high-dose diuretic therapy combined with cortisone. After a month, the patient was eupneic and the anasarca had resolved. We decided gradually to reduce the steroid and diuretic therapy. After ten days of the reduction, the patient began to re-present the same symptoms after treatment with gemcitabine. Corticosteroid therapy was restored with rapid clinical benefit and decreased pro-BNP after a week of treatment. After two years, the disease returned. As a first line treatment, it was decided to use nab-paclitaxel 100 mg/m2 weekly. After two doses, followed by approximately 14 days of treatment, the patient developed acute respiratory distress syndrome. The clinical suspicion was a relapse of capillary leak syndrome and treatment with a high-dose diuretic (furosemide 250 mg daily) was started combined with cortisone (40 mg methylprednisolone). The patient showed a progressive clinical benefit.

**Conclusions:**

In patients treated with gemcitabine and nab-paclitaxel who experience a sudden onset of diffuse edema with respiratory distress, capillary leak syndrome should be suspected. Immediate treatment with corticosteroids may be life-saving.

## Background

Gemcitabine is an antimetabolite pyrimidine, active mainly on tumours of the pancreas, lung (excluding small cell), ovary, breast and bladder [1,2]. Gemcitabine is generally well-tolerated by patients. It can cause pulmonary toxicity in rare cases [3]. The literature also reports cases of thrombotic microangiopathy related to treatment with gemcitabine [4], along with capillary leak syndrome. Nab-paclitaxel was well-tolerated. Non-hematological toxicities were generally mild with grade 1–2 nausea, anorexia, hypocalcemia and vomiting. Grades 3–4 of neutropenia, neutropenic fever, and anemia occurred in 32%, 1% and 11% of patients, respectively [5]. There are no cases of capillary leak syndrome documented in the literature from the use of this drug.

## Case presentation

**Figure 1:**
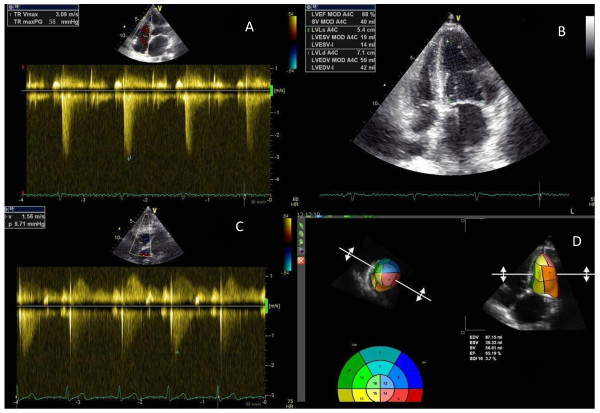
Transthoracic cardiac ecocolordoppler showing evidence of pulmonary hypertension (A-B) and normal pulmonary hypertension and ejection fraction (C-D).

**Figure 2:**
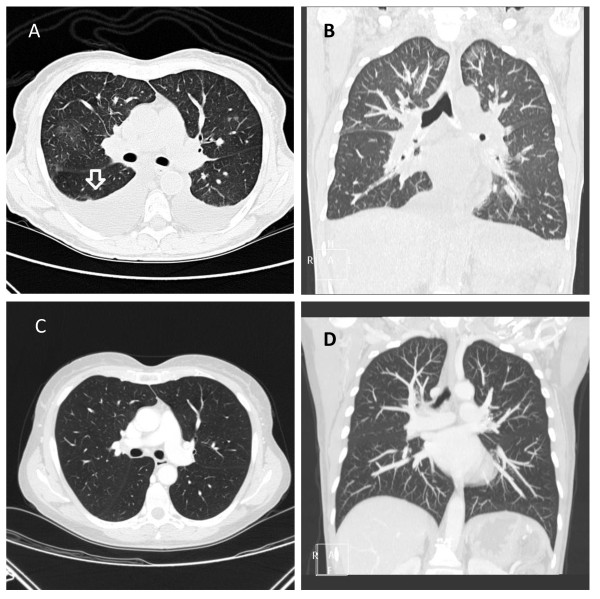
Chest CT scan showing evidence of pulmonary edema (A, axial plane – B, coronal plane) and disappearance of pleural effusion and pulmonary edema (C-D).

A 65-year-old patient underwent a pancreatectomy in January 2010 for ductal carcinoma (pT3 N0 M0, stage IIa). Medical history: in 1994, she underwent an operation for ovarian cancer and was treated with cisplatin, cyclophosphamide and doxorubicin. The treatment was well-tolerated, with no specific toxicity. For the pancreatic cancer, she received adjuvant chemotherapy with gemcitabine (1000 mg/m2 days 1,8,15 every 28 days) from March 2010 to September 2010. During the adjuvant treatment the patient took insulin, aldactone 100 mg, dilatrend and cardioaspirin. Chemotherapy was well-tolerated. Seven days after the last cycle, she developed dyspnea associated with orthopnea and cough. An objective examination showed initial anasarca. A transthoracic cardiac ecocolordoppler was performed with evidence of pulmonary hypertension (58 mmHg) (Figure 1A). The ventricular ejection fraction was normal (68%) (Figure 1B). Blood tests during treatment with chemotherapy were always normal; she now showed an increase in creatinine (1.96 mg/dl), pro-BNP (6425 pg/ml), D-Dimer (636 ug/L) and LDH (503 U/L). There was a decrease in total protein (56 g/l) while, one month previously, total protein had been normal. A CT scan showed bilateral pleural effusions, ascites and the appearance of slight thickening of interlobular septa predominantly at the apices and bases, suggestive of edematous imbibition (Figure 2A-B). Capillary leak syndrome was suspected and the patient was started on high-dose diuretic therapy (furosemide 250 mg daily) combined with cortisone (40 mg methylprednisolone). A slow but progressive clinical benefit was seen. After one month, the patient was eupneic and the anasarca had resolved. A transthoracic cardiac ecocolordoppler was performed with evidence of persistent pulmonary hypertension (44 mmHg). Blood tests showed a persistent rise in creatinine (1.61 mg/dl) with decreased pro-BNP (973 pg/ml). We decided gradually to reduce the steroid (methylprednisolone up to 5 mg per day) and diuretic (furosemide 25 mg per day) therapy. After ten days of the reduction, the patient began to re-present the same symptoms after treatment with gemcitabine. Blood tests showed an increase in pro-BNP (4896 pg/ml), associated with a new increase in pulmonary pressure (50 mmHg). Corticosteroid therapy was restored with rapid clinical benefit and decreased pro-BNP after a week of treatment (1969 pg/ml).

After three months of treatment with corticosteroids and diuretics, the pulmonary pressure had returned to normal. During these three months, the diuretic and steroid therapy was gradually reduced, until it was finally halted. In April 2011, the patient was asymptomatic with normal pulmonary pressure (9.7 mmHg) (Figure 1C).The ventricular ejection fraction was normal (Figure 1D). The pro-BNP and D-dimer levels had normalised, while there was still mild renal insufficiency (1.4). A chest CT scan revealed the disappearance of pleural effusion and pulmonary oedema (Figure 2C-D).

**Figure 3:**
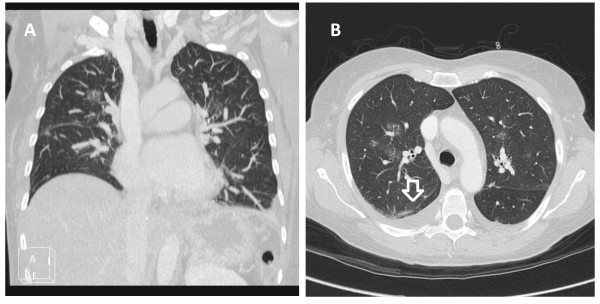
Chest CT scan showing evidence of pulmonary edema (A-B).

Three years after the onset of pain in the right hip, a CT scan of the thorax and abdomen was performed, with evidence of peritoneal carcinomatosis. As a first-line treatment, it was decided to start nab-paclitaxel 100 mg/m2 weekly. After two doses, followed by approximately 14 days of treatment, the patient developed acute respiratory distress syndrome. A CT scan showed bilateral pleural effusions, ascites and the appearance of slight thickening of interlobular septa predominantly at the apices and bases, likely to be oedematous imbibitions (Figure 3A-B). A transthoracic cardiac ecocolordoppler was performed with evidence of pulmonary hypertension (58 mmHg). The ventricular ejection fraction was normal (79%).

Blood tests during treatment with chemotherapy were always normal; she now showed an increase in pro-BNP (4253 pg/ml), D-Dimer (328 ug/L) and LDH (563 U/L).

A relapse of capillary leak syndrome was suspected and the patient was started with high-dose diuretic therapy (furosemide 250 mg daily) combined with cortisone (40 mg methylprednisolone). The patient showed a progressive clinical benefit and, after one week, was eupneic. A transthoracic cardiac ecocolordoppler was performed with evidence of the resolution of pulmonary hypertension (26 mmHg). Blood tests showed a decreased pro-BNP (324 pg/ml). For the patient, it was decided to resume nab-paclitaxel with prophylactic cortisone. After two doses, the pro-BNP had increased to 1500 pg/ml. The patient was asymptomatic. It was decided to discontinue the drug.

## Conclusions

Gemcitabine and nab-paclitaxel are generally well-tolerated but on rare occasions they may have particular toxicity. This toxicity is documented in the literature [6,7]. It usually occurs after a certain number of cycles but in some cases may occur after a single cycle [8,9]. The first case of gemcitabine and non-cardiogenic pulmonary oedema is documented by Pavlakis et al. They presented three cases. Two patients died and the third patient had a good response from cortisone [8]. Conversely, no case of capillary leak syndrome has been reported in literature with nab-paclitaxel.

The peculiarity of this case is that the patient suffered two similar episodes of capillary leak syndrome with two different drugs. The patient saw a benefit with the administration of cortisone. Furthermore, when we reduced the cortisone, her symptoms relapsed. The symptoms disappeared after she has taken the initial dose of cortisone.

Capillary leak syndrome is a systemic process characterised by the hyperpermeability of the endothelium [10]. In this disease there is a rapid extravasation of plasma from the intravascular compartment to the extravascular compartment. This is the cause of the symptoms in patients. The pathophysiology of the damage is not known. A predictor, although a little sensitive, of capillary leak syndrome is the decrease of albumin [11]. This syndrome was described after snake bites [12] in patients with sepsis [13,14] and in patients treated with interleukin-2 [15] and taxotere [16]. Endothelial injury caused a loss of production of nitric oxide (vasodilator). The lack of production of nitric oxide probably caused the increase in pulmonary artery pressure, as caused by vasoconstriction. Pulmonary hypertension led to an overload of the right ventricle; from the laboratory point of view, that caused the increase in pro-BNP.

**Figure 4:**
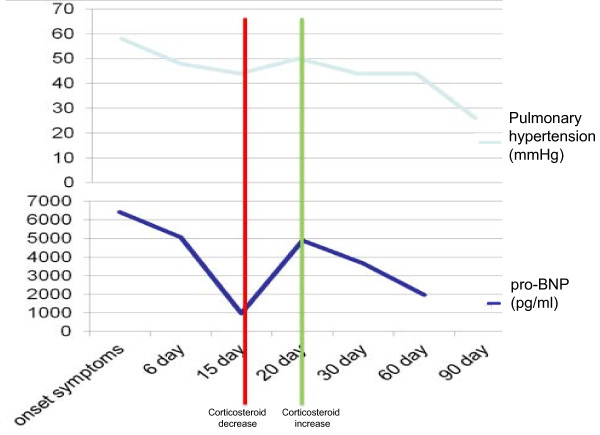
Variation in pro-BNP levels and correlation with pulmonary hypertension.

Our case shows that the variation of pro-BNP is correlated with the clinical patient (Figure 4). During the first remission of symptoms, the pro-BNP decreased to 973. When the corticosteroid was reduced, there was a resurgence of the symptoms with an increase of the pro-BNP. A high dose of cortisone was reintroduced with resolution of the clinical picture and concomitant progressive reduction of the pro-BNP. The trend of pro-BNP levels also followed pulmonary hypertension, suggesting that the lung hypertension was the cause of the appearance of cardiac dysfunction (Figure 4).

Similarly, during the second episode, the pro-BNP followed the pulmonary hypertension. As a differential diagnosis we considered thrombotic microangiopathy, but the clinical and laboratory tests excluded this hypothesis. Furthermore, a diagnosis of vasculitis was unlikely as the indices of inflammation were negative (VES and PCR); there were also high levels of rheumatoid factor and cryoglobulins, and hypocomplementemia was not present. This confirms the direct damage caused by the drug and the absence of immune-mediated damage.

In conclusion, in patients treated with gemcitabine and nab-paclitaxel, the sudden onset of diffuse oedema with respiratory distress tends to suggest this syndrome. Immediate treatment with corticosteroids may save the life of the patient but it is not effective in preventing the syndrome.

## Consent

Written informed consent was obtained from the patient for publication of this case report and accompanying images.

## Competing interests

The authors declare that they have no competing interests.

## Authors’ contributions

ACG treated and observed the patient, performed the literature research and drafted the manuscript. MA observed the patient. DO performed the CT scans, MAB, AL, SC, DA, ET and GLF revised the manuscript. All authors read and approved the final manuscript.

## Pre-publication history

The pre-publication history for this paper can be accessed here:

http://www.biomedcentral.com/1471-2407/13/542/prepub
